# Therapeutic Levels of Acetaminophen Causing AKI: A Case Report and Literature Review

**DOI:** 10.1002/ccr3.70138

**Published:** 2025-01-22

**Authors:** Hamnah Tayyab, Lavanya Dhondapati, Ashraf Ullah, Mohammed Dheyaa Marsool Marsool, Singh Jagmeet, Khabab Abbasher Hussien Mohamed Ahmed

**Affiliations:** ^1^ Guthrie/Robert Packer Hospital Sayre Pennsylvania USA; ^2^ Translational Liver Research Centre Beth Israel Deaconess Boston Massachusetts USA; ^3^ Internal Medicine, Al‐Kindy College of Medicine University of Baghdad Baghdad Iraq; ^4^ Faculty of Medicine University of Khartoum Khartoum Sudan

**Keywords:** acetaminophen, acute kidney injury, adverse drug reaction, paracetamol, therapeutic levels, toxicity

## Abstract

Our case highlights the importance of being aware of the potential renal complications that can occur with acetaminophen, even when used at dosages slightly above the recommended therapeutic dose.

**Abbreviations**
AIN: acute interstitial nephritis
AKI: acute kidney injury
ALP: alkaline phosphatase
ALT: alanine aminotransferase
AST: aspartate aminotransferase
ATN: acute tubular necrosis
CKD: chronic kidney disease
INR: International Normalized Ratio
NAPQI: *N*‐acetyl‐*p*‐benzoquinone imine
NSAIDs: nonsteroidal anti‐inflammatory drugs
PMH: Prior Medical History

## 1 Introduction

The season of colds, coughs, and flu is a good time to review the hazards associated with acetaminophen, the drug that relieves pain and fever and is found in many over‐the‐counter medications, including Tylenol. It is a common ingredient included in several non‐prescription drugs designed to relieve pain and lower body temperature [1]. Although acetaminophen is generally regarded as a safe and effective drug when taken in approved amounts, consuming more than 4 g (4000 mg) in a day is considered dangerous and can result in significant liver damage and other severe health consequences [2].

Acetaminophen is one of the most frequently used analgesics for both acute and chronic pain management [1, 3]. However, the risk of toxicity becomes a concern when the drug is consumed in amounts greater than the recommended dosage. Overdosing on acetaminophen is a leading cause of acute liver failure in the United States and the United Kingdom [4]. While the hepatotoxic effects of acetaminophen overdose are well documented, it is still unclear how often kidney damage occurs when taking normal amounts of the medication.

Therapeutic doses of acetaminophen are generally regarded as safe for the kidneys. However, in approximately 1%–2% of cases, even acute intoxication at these levels can result in renal damage. This raises questions about the potential nephrotoxic effects of acetaminophen, particularly in healthy individuals without pre‐existing liver conditions. The increased excretion of the drug and its toxic metabolite, *N*‐acetyl‐*p*‐benzoquinone imine (NAPQI), characterizes the pathogenesis of acetaminophen‐induced kidney injury [5]. This leads to subendothelial damage and tubular ischemia, ultimately resulting in acute tubular necrosis.

In this case report, we describe the medical condition of a 49‐year‐old man who experienced sudden renal damage after taking the recommended amounts of acetaminophen. Although the patient had therapeutic plasma acetaminophen levels and just a mild liver injury, he had severe kidney damage, resulting in a need for dialysis.

## 2 Case Presentation

### 2.1 History/Examination

A 49‐year‐old male with no prior medical history (PMH) presented to the emergency department with a 3‐day history of abdominal pain, malaise, generalized body aches, nausea, vomiting, and oliguria with brown‐tinged urine that had been ongoing for 1 day. The patient reported taking two tablets of Tylenol (650 mg each) and TheraFlu (containing 325 mg of acetaminophen) 30 mL every 4 h for the past 3 days to manage his symptoms. At admission, the patient reported that his last dose of acetaminophen was taken approximately 4 h before the blood sample was collected for acetaminophen level measurement.

### 2.2 Differential Diagnosis

**FIGURE 1:**
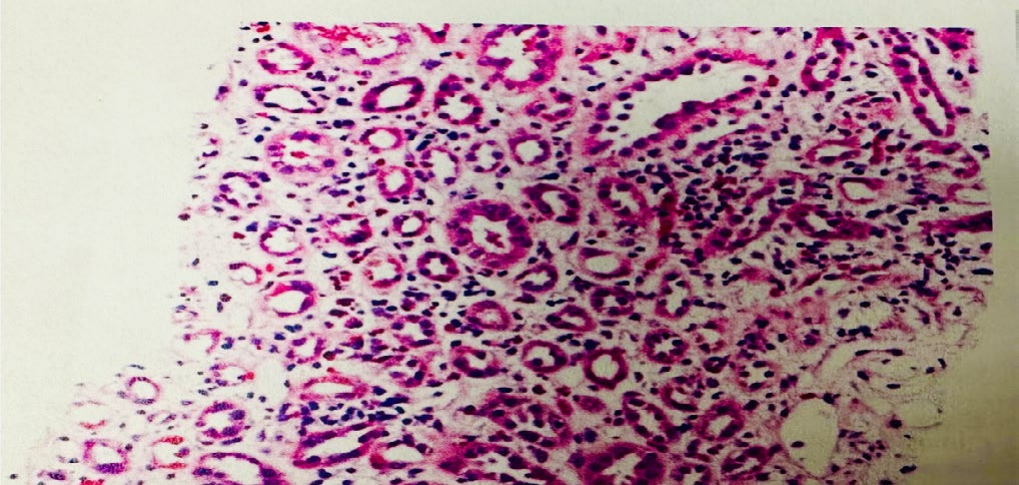
Kidney biopsy showing acute tubulointerstitial nephritis with eosinophil, moderate acute tubular epithelial cell injury, and osmotic tubulopathy. There is also moderate arteriosclerosis.

Upon admission, laboratory investigations revealed the following results (Table 1): therapeutic acetaminophen levels at 5.3 μg/mL, significantly elevated creatinine at 6.7 mg/dL (baseline creatinine was 0.9 mg/dL), elevated liver enzymes with AST at 7000 U/L, ALT at 5000 U/L, and ALP at 163 U/L. The markedly elevated AST and ALT levels (AST: 7000 U/L, ALT: 5000 U/L) indicate significant hepatocellular injury. While the patient did not develop fulminant hepatic failure, the possibility of hepatorenal syndrome contributing to the acute kidney injury cannot be entirely excluded. However, the rapid improvement in liver enzymes and the histopathological findings of acute tubular necrosis suggest a direct nephrotoxic effect of acetaminophen and its metabolites. The hepatitis panel was unremarkable, bilirubin was elevated at 2.7 mg/dL, and prothrombin time was slightly prolonged at 1.3. A vasculitis workup was also unremarkable. Urine microscopy showed no signs of inflammatory pathology. A kidney biopsy later confirmed the presence of acute interstitial nephritis (AIN) and acute tubular necrosis (ATN) (Figure 1).

**TABLE 1:** Laboratory investigations.

Test	Result	Reference range
Acetaminophen level	5.3 μg/mL	10–30 μg/mL
Creatinine	6.7 mg/dL	0.67–1.17 mg/dL
AST	7000 U/L	≤ 40 U/L
ALT	5000 U/L	5–41 U/L
ALP	163 U/L	40–129 U/L
Lactic acid	1.7 mmol/L	0–5‐2.2 mmol/L
Bilirubin	Total 2.7 mg/dL	0.00–1.10 mg/dL
	Direct 1.5 mg/dL	0.0–0.3 mg/dL
Prothrombin time	1.3 (INR)	0.88–1.13
Hepatitis panel		
Anti‐HAV IgM	Non‐reactive	
HBsAg	Non‐reactive	
Anti‐HBs	Non‐reactive	
Anti‐HBc	Non‐reactive	
Anti‐HCV	Non‐reactive	
Antibody screening		
ASMA	Negative	
ANA	Negative	
Anti‐GBM	< 1.0	
ANCA	< 1.0	

Abbreviations: ALP, alkaline phosphatase; ALT, alanine aminotransferase; ANA, antinuclear antibodies; ANCA, antinuclear cytoplasmic antibodies; anti‐GBM, anti–glomerular basement membrane antibodies; ASMA, anti–smooth muscle antibodies; AST, aspartate aminotransferase.

**FIGURE 2:**
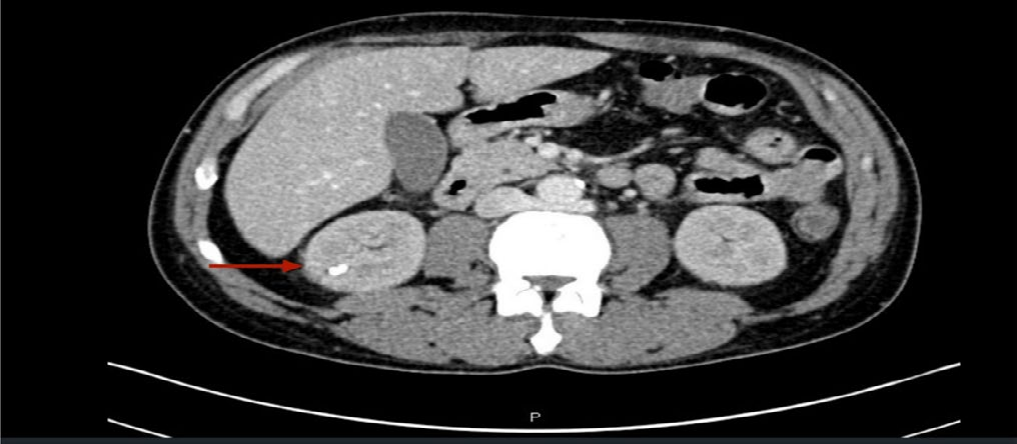
Computed tomography was unremarkable for any cortical or medullary damage.

Imaging studies included a CT scan of the chest, abdomen, and pelvis, which revealed numerous renal calculi measuring 5 mm each, with no evidence of hydronephrosis. The pancreas and spleen appeared unremarkable. A renal ultrasound did not show any significant pathology (Figure 2).

## 3 Conclusion and Results

In the emergency department, the patient was treated with fluid resuscitation using Ringer's lactate solution. Due to concerns about the adverse side effects of acetaminophen, even at therapeutic levels, the patient was started on an *N*‐acetylcysteine protocol.

During his hospital stay, the patient's liver function tests showed gradual improvement. However, his kidney function continued to deteriorate despite aggressive fluid resuscitation and diuretic therapy. His creatinine levels continued to rise, reaching a peak of 21.5 mg/dL, accompanied by a significant decrease in urine output. Given the severity of his renal impairment, dialysis was initiated.

The patient underwent four sessions of dialysis, after which there were signs of renal recovery. His creatinine levels began to decline, and his urine output improved. At the time of discharge, his creatinine level had decreased to 5.7 mg/dL (Table 2). Follow‐up 1 month later showed a further decrease in creatinine to 2.5 mg/dL, eventually returning to his baseline level of 0.9 mg/dL.

**TABLE 2:** Creatinine trends and dialysis response.

Day	Creatinine levels (mg/dL)	Intervention
1	6.7	Admission, fluids initiated
2	6.8	Continued fluids
3	15	Dialysis initiated
4	16.5	Dialysis session 1
5	19.2	Dialysis session 2
6	21.5	Dialysis session 3
7	16.1	Post‐dialysis 1
8	17.5	Post‐dialysis 2
9	12.6	Post‐dialysis 3
10	9.6	Post‐dialysis 4
Discharge	5.7	Improvement noted
1‐month later	2.5	Continued improvement
Final follow‐up	0.9	Baseline recovery

Analgesic nephropathy was first introduced in the 1950s, but the evidence of the nephrotoxicity of analgesic drugs, acetaminophen, aspirin, and other nonsteroidal anti‐inflammatory drugs (NSAIDs) is scanty and inconsistent. Ingestion of excessive amounts of acetaminophen can lead to AKI due to acute tubular necrosis at the proximal tubule. The excess amount of acetaminophen can indirectly affect the kidneys by the formation of the toxic metabolite NAPQI, leading to oxidative stress and causing acute kidney injury over time. Additionally, long‐term use can cause CKD, which can lead to end‐stage renal disease due to papillary necrosis or chronic interstitial fibrosis. When used at therapeutic dosages, acetaminophen can still cause kidney toxicity, particularly in individuals who have contributing factors leading to glutathione depletion such as persistent alcohol consumption, malnutrition, or fasting. Therapeutic doses of paracetamol have been found to cause a small but significant amount of apoptosis in cultivated tubular epithelial cells, leading to AIN, while the exact mechanism is unknown.

## 4 Discussion

Acetaminophen is one of the most widely available and used without prescription drugs and is considered safe when used at therapeutic doses of < 4 g per day [1, 2]. However, toxicity can arise from dosing mistakes, accidental ingestions by children, and intentional overdoses for self‐harm. Acetaminophen overdose is a leading cause of acute liver failure in the United States and United Kingdom [4]. In general, renal insufficiency affects about 2%–10% of patients who experience an acetaminophen overdose.

There are only a few case reports and observational studies of acute renal failure at therapeutic doses of acetaminophen without concomitant severe liver injury mostly reported in children and adolescents [6, 7, 8, 9, 10]. Our patient had been consuming two tablets of Tylenol (650 mg each) and TheraFlu (containing 325 mg of acetaminophen) every 4 h, totaling 5850 mg per day, which exceeds the recommended maximum dose of 4000 mg. Kidney injury is due to large amounts of excretion of acetaminophen and NAPQ1, the toxic metabolite of acetaminophen, which causes subendothelial damage and tubular ischemic change and then progresses to acute tubular necrosis [5].

At therapeutic doses, acetaminophen can be toxic to the kidneys in patients with depleted glutathione levels due to chronic alcohol consumption, starvation, or fasting, or in those taking drugs that activate P‐450 microsomal oxidase enzymes, such as anticonvulsants [1, 5]. Our patient has no such history of heavy alcohol consumption or use of any drugs.

Acute renal failure typically begins 2–5 days after acetaminophen ingestion, with peak serum creatinine levels occurring 3–16 days later, averaging around 7.3 days post‐overdose [2, 5]. This was the case with our patient; the liver function tests improved during the first few days of the hospital stay, but kidney function kept deteriorating, reaching peak levels in almost 1 week after which dialysis was initiated. The cumulative excessive intake of acetaminophen over several days likely led to accumulative toxicity, contributing to the development of acute kidney injury in this patient.

The management of acetaminophen‐related nephrotoxicity is largely *N*‐acetylcysteine as most patients have concomitant hepatotoxicity and symptomatic management of complications [11]. In most cases of nephrotoxicity, without acute liver failure, the acute renal failure is reversible [5, 6]. In the case of our patient, renal function started improving after four sessions of dialysis. His renal function completely returned to normal after his discharge from the hospital.

## Author Contributions

**Hamnah Tayyab:** conceptualization, data curation, formal analysis, funding acquisition, investigation, methodology, project administration, resources, software, supervision, validation, visualization, writing – original draft, writing – review and editing. **Lavanya Dhondapati:** conceptualization, data curation, formal analysis, funding acquisition, investigation, methodology, project administration, validation, visualization, writing – original draft, writing – review and editing. **Ashraf Ullah:** conceptualization, data curation, formal analysis, funding acquisition, investigation, methodology, validation, visualization, writing – original draft, writing – review and editing. **Mohammed Dheyaa Marsool Marsool:** conceptualization, supervision, validation, visualization, writing – original draft, writing – review and editing. **Singh Jagmeet:** conceptualization, validation, visualization, writing – original draft, writing – review and editing. **Khabab Abbasher Hussien Mohamed Ahmed:** conceptualization, validation, visualization, writing – original draft, writing – review and editing.

## Consent

Written informed consent was obtained from the patient to publish this report in accordance with the journal's patient consent policy.

## Conflicts of Interest

The authors declare no conflicts of interest.

## Data Availability

The data that support the findings of this study are available from the corresponding author upon reasonable request.
